# *CCDN1* rs603965 polymorphism may serve as a genetic biomarker of brain tumor: A meta‐analysis of 5,769 subjects

**DOI:** 10.1002/mgg3.655

**Published:** 2019-04-10

**Authors:** Jiarong Lan, Min Li, Haifeng Wang

**Affiliations:** ^1^ Department of Nephrology Huzhou Hospital of Traditional Chinese Medicine Affiliated Zhejiang University of Traditional Chinese Medicine Huzhou Zhejiang China; ^2^ Department of Endocrinology Huzhou Hospital of Traditional Chinese Medicine Affiliated Zhejiang University of Traditional Chinese Medicine Huzhou Zhejiang China; ^3^ Department of Neurosurgery Ningbo No. 1 Hospital Ningbo Zhejiang China

**Keywords:** brain tumor, cyclin D1 (CCND1), glioma, meta‐analysis, rs603965 polymorphism

## Abstract

**Introduction:**

Some studies already tried to assess the associations between cyclin D1 (*CCND1*) polymorphisms and brain tumor. However, the results of these studies were not consistent. Thus, we performed the present meta‐analysis to explore the relationship between *CCND1* polymorphisms and brain tumor in a larger pooled population.

**Methods:**

PubMed, Web of Science, Embase, and CNKI were searched for related articles. Odds ratios (ORs) and 95% confidence intervals (CIs) were calculated to assess the potential associations.

**Results:**

Totally nine studies with 5,769 subjects were analyzed. A significant association with brain tumor susceptibility was observed for the rs603965 polymorphism in GG versus GA + AA (dominant comparison, *p *=* *0.003, OR = 0.72, 95% CI 0.57–0.89, *I* ^2^ = 64%), AA versus GG + GA (recessive comparison, *p *=* *0.004, OR = 1.46, 95% CI 1.13–1.88, *I* ^2^ = 67%), and G versus A (allele comparison, *p *=* *0.0004, OR = 0.77, 95% CI 0.66–0.89, *I* ^2^ = 66%) in overall population. Further subgroup analyses by ethnicity yielded similar positive results in both Asians and Caucasians. Moreover, in stratified analyses by type of disease, we noticed that the rs603965 polymorphism was significantly associated with the susceptibility to glioma, but such positive results were not detected in pituitary adenoma or meningioma. Additionally, a significant association with tumor grade was also observed for the rs603965 polymorphism in G versus A (allele comparison, *p *=* *0.02, OR = 0.74, 95% CI 0.59–0.95, *I* ^2^ = 26%).

**Conclusions:**

Our findings suggested that *CCND1* rs603965 polymorphism may serve as a potential genetic biomarker of brain tumor, especially for glioma.

## 1 INTRODUCTION

Brain tumor refers to cancer that originates from the brain, and it accounts for 1.8% of new cancers and 2.3% of cancer related deaths all over the world (Siegel, Miller, & Jemal, 2017). Despite rapid progress in chemotherapy, radiation therapy, and minimally invasive surgical treatment achieved in the past few decades, the 5‐year survival rate of brain tumor is still extremely low (McNeill, 2016; Zhang et al., 2017). Therefore, early diagnosis is of greatest importance, and identify its potential biomarkers is vital for further improving the prognosis of patients with brain tumor.

Although the exact etiologies of brain tumor are still poorly understood, the obvious family aggregation tendency of brain tumor suggests that inherited factors are involved in its development (Lapointe, Perry, & Butowski, 2018; Suvà & Louis, 2013). Cyclin D1 (CCND1) is encoded by the *CCND1* gene located on chromosome 11q13. It controls the G1 ‐ S phase transition of the cell cycle and plays a crucial role in the regulation of cell proliferation and differentiation (Casimiro, Velasco‐Velázquez, Aguirre‐Alvarado, & Pestell, 2014; Qie & Diehl, 2016). Genetic variations in the *CCND1* gene may lead to alternations in gene expression or changes in CCND1 protein structure, which may subsequently affect biological functions of CCND1 and ultimately impact individual susceptibility to multiple malignancies including brain tumor.

Recently, some studies already tried to assess the associations between *CCND1* polymorphisms and brain tumor. However, the results of these studies were not consistent (Cander et al., 2012; Chen et al., 2012; Gazioglu et al., 2007; Liu et al., 2015). Previous studies failed to reach a consensus regarding associations between *CCND1* polymorphisms and brain tumor partially because of their relatively small sample sizes. Thus, we performed the present meta‐analysis to explore the relationship between *CCND1* polymorphisms and brain tumor in a larger pooled population.

## 2 MATERIALS AND METHODS

### 2.1 Literature search and inclusion criteria

We followed the Preferred Reporting Items for Systematic Reviews and Meta‐analyses (PRISMA) guideline when conducting this meta‐analysis (Moher, Liberati, Tetzlaff, & Altman, 2009). Four databases (PubMed, Web of Science, Embase, and CNKI) were searched for potentially related articles using the following terms: “cyclin D1”, “CCND1”, “ BCL1”, “PRAD1”, “U21B31”, “D11S287E”, “polymorphism”, “variant”, “mutation”, “variation”, “brain tumor”, “brain neoplasm”, “brain cancer”, “glioma”, “glioblastoma”, “astrocytoma”, “meningioma”, “pituitary adenoma”, and “prolactinoma”. The reference lists of all retrieved articles were also screened for other potentially related studies.

Included studies of this meta‐analysis must meet all the following criteria: (a) study on associations between *CCND1* polymorphism and susceptibility to brain tumor/biological characteristics of brain tumor; (b) providing genotypic distribution of investigated polymorphisms in cases and controls; (c) full text in English or Chinese available. Studies were excluded if one of the following criteria was fulfilled: (a) not about *CCND1* polymorphism and brain tumor; (b) insufficient data to estimate associations between *CCND1* polymorphism and brain tumor; (c) reviews, editorials, or comments. In the case of duplicate reports by the same authors, we only included the study with the largest sample size.

### 2.2 Data extraction and quality assessment

From eligible studies, we extracted the following information: name of the first author, year of publication, country and ethnicity of participants, sample size, and the genotypic distribution of *CCND1* polymorphism in cases and controls. The probability value (*p* value) of Hardy–Weinberg equilibrium (HWE) was also calculated.

We used the Newcastle‐Ottawa scale (NOS) to evaluate the quality of eligible studies (Stang, 2010). The NOS has a score range of zero to nine, and studies with a score of more than seven were thought to be of high quality.

Two reviewers conducted data extraction and quality assessment independently. When necessary, we wrote to the corresponding authors for extra information. Any disagreement between two reviewers was solved by discussion until a consensus was reached.

### 2.3 Statistical analyses

In this study, statistical analyses were performed by using Review Manager Version 5.3.3. Pooled odds ratios (ORs) and 95% confidence intervals (CIs) were calculated to estimate potential associations between *CCND1* genetic polymorphism and brain tumor in dominant, recessive, overdominant and allele genetic models, and a *p* value of 0.05 or less was defined as statistically significant. Between‐study heterogeneities were evaluated by using the *I* ^2^ statistic. Random‐effect models (REMs) would be used for analyses if *I* ^2^ was greater than 50%. Otherwise, analyses would be conducted with fixed‐effect models (FEMs). Subgroup analyses were subsequently carried out by type of disease and ethnicity of participants. Stabilities of synthetic results were tested in sensitivity analyses. Publication biases were assessed by funnel plots.

## 3 RESULTS

### 3.1 Characteristics of included studies

**Figure 1:**
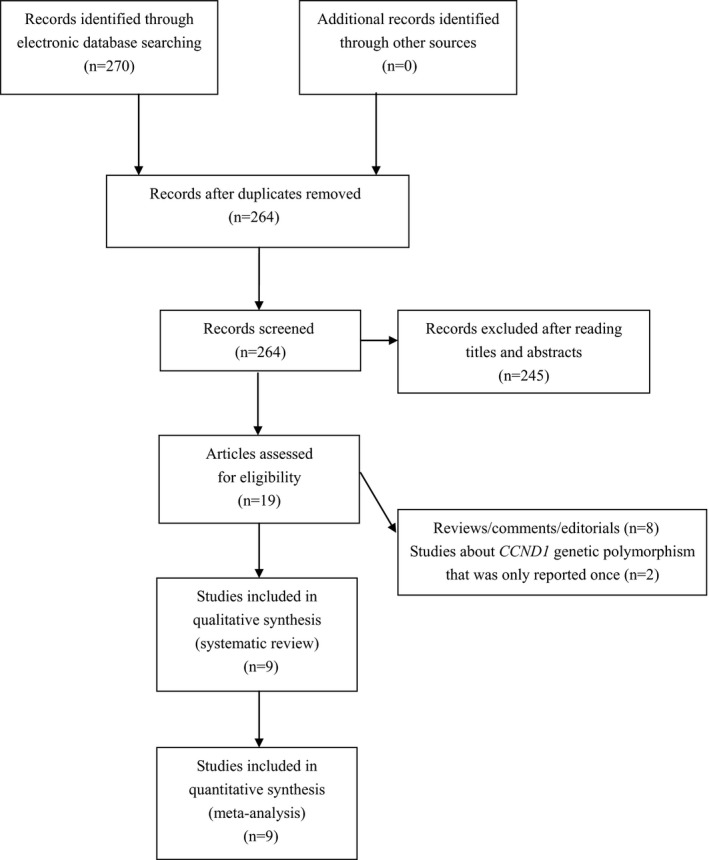
Flowchart of study selection for this study

The literature search procedure was shown in Figure 1. Totally 270 articles were found by using our searching strategy. After excluding irrelevant and duplicate articles, 19 articles were retrieved for further evaluation. Another 10 articles were subsequently excluded after reading the full text. Ultimately, a total of nine eligible studies involving 2,079 cases and 3,690 controls were enrolled in analyses (see Figure 1). Characteristics of included studies were summarized in Table 1.

**Table 1:** The characteristics of included studies

First author, year	Country	Ethnicity	Type of disease	Sample size	Genotypes (GG/GA/AA)		*p*‐value for HWE	NOS score
					Cases	Controls		
Tumor susceptibility								
Cander et al., 2012	Turkey	Caucasian	Pituitary adenoma	113/108	15/58/40	41/44/23	0.092	8
Chen et al., 2012	China	Asian	Glioma	170/170	41/64/65	47/80/43	0.447	8
Fan 2010	China	Asian	Glioma	161/89	33/85/43	19/49/21	0.337	8
Gazioglu et al., 2007	Turkey	Caucasian	Pituitary adenoma	130/129	10/90/30	29/64/36	0.956	7
Liu et al., 2015	China	Asian	Glioma	167/180	61/47/59	82/65/33	0.003	8
Rajaraman et al., 2007	USA	Mixed	Glioma	374/528	105/196/73	183/245/100	0.266	7
Rajaraman et al., 2007	USA	Mixed	Meningioma	151/528	47/63/41	183/245/100	0.266	7
Rajaraman et al., 2007	USA	Mixed	Acoustic neuroma	71/528	25/36/10	183/245/100	0.266	7
Simpson et al., 2001	UK	Caucasian	Pituitary adenoma	294/414	97/120/77	132/206/76	0.780	8
Yang et al., 2017	China	Asian	Glioma	350/706	162/142/46	436/240/30	0.674	8
Zeybek et al., 2013	Turkey	Caucasian	Glioma	57/155	13/26/18	40/73/42	0.471	8
Zeybek et al., 2013	Turkey	Caucasian	Meningioma	41/155	11/20/11	40/73/42	0.471	8
Tumor grade					High	Low		
Chen et al., 2012	China	Asian	Glioma	102/68	22/42/38	19/22/27	0.005	8
Fan 2010	China	Asian	Glioma	53/36	9/33/11	10/16/10	0.505	8
Simpson et al., 2001	UK	Caucasian	Pituitary adenoma	122/172	36/42/44	63/76/33	0.246	8
Tumor size					Macro	Micro		
Cander et al., 2012	Turkey	Caucasian	Pituitary adenoma	61/52	9/30/22	6/28/18	0.321	8
Gazioglu et al., 2007	Turkey	Caucasian	Pituitary adenoma	49/47	4/34/11	4/34/9	0.001	7
Tumor Invasiveness					Invasive	Non‐invasive		
Cander et al., 2012	Turkey	Caucasian	Pituitary adenoma	28/85	5/13/10	10/45/30	0.265	8
Gazioglu et al., 2007	Turkey	Caucasian	Pituitary adenoma	32/98	2/20/10	8/70/20	<0.001	7

HWE: Hardy–Weinberg equilibrium; NOS: Newcastle‐ottawa scale.

### 3.2 CCND1 rs603965 polymorphism and susceptibility to brain tumor

Totally 5,769 subjects were enrolled in pooled analyses. A significant association with brain tumor susceptibility was observed for the rs603965 polymorphism in GG versus GA + AA (dominant comparison, *p *=* *0.003, OR = 0.72, 95% CI 0.57–0.89, *I* ^2^ = 64%), AA versus GG + GA (recessive comparison, *p *=* *0.004, OR = 1.46, 95% CI 1.13–1.88, *I* ^2^ = 67%), and G versus A (allele comparison, *p *=* *0.0004, OR = 0.77, 95% CI 0.66–0.89, *I* ^2^ = 66%) in overall population. Further subgroup analyses by ethnicity yielded similar positive results in both Asians (dominant, recessive, and allele comparisons) and Caucasians (recessive comparison). Moreover, in stratified analyses by type of disease, we noticed that the rs603965 polymorphism was significantly associated with the susceptibility to glioma, but such positive results were not detected in pituitary adenoma or meningioma (see Table 2).

**Table 2:** Results of overall and subgroup analyses for *CCND1* G870A polymorphism and brain tumor

Population	Sample size	Dominant comparison			Recessive comparison			Overdominant comparison			Allele comparison		
		*p* value	OR (95%CI)	*I* ^2^ statistic (%)	*p* value	OR (95%CI)	*I* ^2^ statistic (%)	*p* value	OR (95%CI)	*I* ^2^ statistic (%)	*p* value	OR (95%CI)	*I* ^2^ statistic (%)
Tumor susceptibility													
Overall	2,079/3,690	**0.003**	**0.72 (0.57**–**0.89)**	64	**0.004**	**1.46 (1.13**–**1.88)**	67	0.72	1.04 (0.84–1.28)	66	**0.0004**	**0.77 (0.66**–**0.89)**	66
Pituitary adenoma	537/651	0.13	0.44 (0.15–1.27)	90	0.23	1.36 (0.82–2.26)	67	0.48	1.32 (0.61–2.83)	89	0.06	0.70 (0.48–1.02)	77
Glioma	1,279/1,828	**<0.000**	**0.67 (0.58**–**0.79)**	13	**0.01**	**1.69 (1.13**–**2.54)**	67	0.89	0.98 (0.76–1.26)	60	**0.002**	**0.72 (0.59**–**0.88)**	68
Meningioma	192/683	0.50	0.89 (0.63–1.26)	0	0.06	1.42 (0.98–2.06)	12	0.42	0.87 (0.63–1.21)	0	0.14	0.84 (0.67–1.06)	19
Asian	848/1,145	**<0.0001**	**0.64 (0.52**–**0.77)**	35	**0.0005**	**2.10 (1.39**–**3.19)**	63	0.55	0.89 (0.62–1.29)	70	**0.003**	**0.66 (0.52**–**0.82)**	60
Caucasian	635/961	0.11	0.60 (0.32–1.13)	81	**0.02**	**1.34 (1.06**–**1.69)**	42	0.49	1.18 (0.73–1.91)	78	0.06	0.78 (0.60–1.01)	62
Tumor grade													
Overall	277/276	0.06	0.69 (0.47–1.01)	0	0.64	1.21 (0.55–2.65)	74	0.63	1.18 (0.59–2.37)	71	**0.02**	**0.74 (0.59**–**0.95)**	26
Asian	155/104	0.14	0.65 (0.36–1.16)	14	0.49	0.83 (0.49–1.41)	0	0.06	1.66 (0.99–2.77)	0	0.66	0.92 (0.65–1.32)	0
Glioma	155/104	0.14	0.65 (0.36–1.16)	14	0.49	0.83 (0.49–1.41)	0	0.06	1.66 (0.99–2.77)	0	0.66	0.92 (0.65–1.32)	0
Tumor size													
Overall	110/99	0.72	1.18 (0.49–2.83)	0	0.71	1.12 (0.61–2.07)	0	0.56	0.84 (0.48–1.49)	0	0.94	0.99 (0.67–1.46)	0
Caucasian	110/99	0.72	1.18 (0.49–2.83)	0	0.71	1.12 (0.61–2.07)	0	0.56	0.84 (0.48–1.49)	0	0.94	0.99 (0.67–1.46)	0
Pituitary adenoma	110/99	0.72	1.18 (0.49–2.83)	0	0.71	1.12 (0.61–2.07)	0	0.56	0.84 (0.48–1.49)	0	0.94	0.99 (0.67–1.46)	0
Tumor invasiveness													
Overall	60/183	0.69	1.21 (0.48–3.07)	0	0.37	1.33 (0.71–2.50)	0	0.28	0.72 (0.39–1.31)	0	0.69	0.92 (0.60–1.40)	0
Caucasian	60/183	0.69	1.21 (0.48–3.07)	0	0.37	1.33 (0.71–2.50)	0	0.28	0.72 (0.39–1.31)	0	0.69	0.92 (0.60–1.40)	0
Pituitary adenoma	60/183	0.69	1.21 (0.48–3.07)	0	0.37	1.33 (0.71–2.50)	0	0.28	0.72 (0.39–1.31)	0	0.69	0.92 (0.60–1.40)	0

OR: odds ratio; CI: confidence interval; NA: not available. The values in bold represent there is statistically significant differences between cases and controls.

### 3.3 CCND1 rs603965 polymorphism and biological characteristics of brain tumor

Totally 868 subjects were enrolled in pooled analyses. A significant association with tumor grade was observed for the rs603965 polymorphism in G versus A (allele comparison, *p *=* *0.02, OR = 0.74, 95% CI 0.59–0.95, *I* ^2^ = 26%). Nevertheless, no any positive results were detected for rs603965 polymorphism when it comes to tumor size or tumor invasiveness (see Table 2).

### 3.4 Sensitivity analyses

Sensitivity analyses were performed to test the effects of individual study on pooled results if the original analysis was based on at least four studies. No any alterations of results were detected in overall and subgroup analyses, which suggested that our findings were statistically stable.

### 3.5 Publication biases

We used funnel plots to evaluate potential publication biases. The shape of funnel plots was symmetry for every comparison, which indicated that our findings were unlikely to be impacted by severe publication biases.

## 4 DISCUSSION

As far as we know, this is so far the most comprehensive meta‐analysis about *CCND1* polymorphism and brain tumor. The pooled results showed that the rs603965 polymorphism was significantly associated with the susceptibility to brain tumor. Moreover, it was also significantly associated with tumor grade. The positive results obtained with the susceptibility to brain tumor were largely driven by the glioma subgroup, yet no statistically significant associations were observed in pituitary adenoma and meningioma subgroups. The stabilities of synthetic results were evaluated by sensitivity analyses, and no alterations of results were observed in any comparisons, which suggested that our findings were statistically stable.

There are several points that worth noting about this meta‐analysis. First, the present meta‐analysis aimed to explore associations between all *CCND1* polymorphisms and brain tumor. However, only the rs603965 polymorphism could be analyzed because this was the only *CCND1* polymorphism that was investigated by multiple different studies. Previous experimental studies found that the rs603965 polymorphism was located at the conjunction of exon 4 and intron 4 of *CCND1* gene, the G to A variation in this locus was associated with a shorter transcription and a 55 amino acids loss of the C‐terminal domain of CCND1 (Jeon, Kim, Jeong, Bae, & Jung, 2018; Vodicka et al., 2018), which may greatly alter the activity of CCND1 and give rise to the development of brain tumor. Second, the etiologies of different types of brain tumor may be somewhat different. Therefore, although it is biologically possible that *CCDN1* rs603965 polymorphism may be implicated in the development of brain tumor, its effects on different types of brain tumor may be different, and this may partially explain the observed heterogeneities in the current meta‐analysis. Third, it is notable that in 2014, Qin et al. (2014) also performed a meta‐analysis to estimate associations between *CCDN1* rs603965 polymorphism and brain tumor. The current meta‐analysis should be considered as a valuable supplementary work to this previous meta‐analysis because of the following two points, (a) Three related studies were published in the last 4 years. Therefore, an update meta‐analysis is warranted and the sample sizes of our analyses were also significantly larger than of previous meta‐analysis, which could significantly reduce the risk of obtaining false positive or false negative results; (b) This meta‐analysis also tried to investigate potential associations between *CCDN1* rs603965 polymorphism and biological characteristics of brain tumor. Although the sample size was still relatively small, the results obtained were quite interesting, and we call on future studies to report related data. Fourth, in the current meta‐analysis, we noticed that studies conducted in Asians were mainly about glioma, while studies conducted in Caucasians were mainly about pituitary adenoma. This phenomenon suggested that the disease spectrum of brain tumor in different ethnicities may be quite different, and the exact reason underlying this phenomenon is also worth investigating. Fifth, the functional significance of the rs603965 polymorphism is well established, yet no significant associations were observed for pituitary adenoma or meningioma. Since the sample sizes of pooled analyses in the current meta‐analysis were still relatively small, it is possible that our study was still not statistically adequate to detect the actual associations between the rs603965 polymorphism and other types of brain tumor. Therefore, further studies with larger sample sizes still need to confirm our findings.

Some limitations of this meta‐analysis should also be noted when interpreting our findings. First, due to lack of raw data, our pooled analyses were based on unadjusted analyses, but we have to admit that failure to perform further adjusted analyses for confounding factors may impact the reliability of our findings (Qian, Zhang, Qian, He, & Li, 2017). Second, grey literatures like research materials that were not formally published in academic journals were not considered to be eligible for analyses in this meta‐analysis since it is hard to evaluate their quality. However, since grey literatures were not analyzed, although funnel plots suggested that severe publication biases were unlikely to be existed in the current meta‐analysis, it is still possible that our findings may be impacted by potential publication biases (Liu & Jiang, 2017). Third, associations between *CCND1* rs603965 polymorphism and brain tumor may also be modified by gene‐environmental interactions. However, most studies did not explore the effects of these potential interactions, which impeded us to conduct relevant analyses accordingly (Montelli Tde et al., 2011). Considering the above mentioned limitations, our findings should be interpreted with caution.

### 4.1 CONCLUSION

In summary, our meta‐analysis suggested that the *CCND1* rs603965 polymorphism may serve as a potential genetic biomarker of brain tumor, especially for glioma. However, further well‐designed studies with larger sample sizes are still warranted to confirm our findings.

## CONFLICT OF INTEREST

The authors declare that they have no conflict of interest.

## AUTHOR CONTRIBUTION

Jiarong Lan and Haifeng Wang conceived of the study, participated in its design. Jiarong Lan and Min Li conducted the systematic literature review. Min Li performed data analyses. Jiarong Lan and Haifeng Wang drafted the manuscript. All authors have read and approved the final manuscript.

## ETHICAL APPROVAL

This article does not contain any studies with human participants or animals performed by any of the authors.

## INFORMED CONSENT

For this type of study formal consent is not required.
